# An AMPK-dependent regulatory pathway in tau-mediated toxicity

**DOI:** 10.1242/bio.022863

**Published:** 2017-08-14

**Authors:** Alessia Galasso, Charles S. Cameron, Bruno G. Frenguelli, Kevin G. Moffat

**Affiliations:** School of Life Sciences, University of Warwick, Coventry CV4 7AL, UK

**Keywords:** Neurodegeneration, Tau, AMPK, Autophagy, *Drosophila*

## Abstract

Neurodegenerative tauopathies are characterised by accumulation of hyperphosphorylated tau aggregates primarily degraded by autophagy. The 5′AMP-activated protein kinase (AMPK) is expressed in most cells, including neurons. Alongside its metabolic functions, it is also known to be activated in Alzheimer's brains, phosphorylate tau, and be a critical autophagy activator. Whether it plays a neurotoxic or neuroprotective role remains unclear. In tauopathies stress conditions can result in AMPK activation, enhancing tau-mediated toxicity. Paradoxically, in these cases AMPK activation does not always lead to protective autophagic responses. Using a *Drosophila in vivo* quantitative approach, we have analysed the impact of AMPK and autophagy on tau-mediated toxicity, recapitulating the AMPK-mediated tauopathy condition: increased tau phosphorylation, without corresponding autophagy activation. We have demonstrated that AMPK binding to and phosphorylating tau at Ser-262, a site reported to facilitate soluble tau accumulation, affects its degradation. This phosphorylation results in exacerbation of tau toxicity and is ameliorated via rapamycin-induced autophagy stimulation. Our findings support the development of combinatorial therapies effective at reducing tau toxicity targeting tau phosphorylation and AMPK-independent autophagic induction. The proposed *in vivo* tool represents an ideal readout to perform preliminary screening for drugs promoting this process.

## Abstract (precis)

**Summary:** Dissection of the impact of AMPK and autophagy on tau-mediated toxicity by using an *in vivo Drosophila* tool as readout to perform preliminary drug screening supported by quantitative analyses.

## INTRODUCTION

The microtubule (MT)-associated protein tau regulates assembly and stability of MTs (Lee et al., 2001). In physiological conditions, tau is in a dynamic equilibrium, on and off the MTs. Under pathological conditions, this equilibrium is perturbed, resulting in an abnormal increase in the aggregation-prone unbound tau fraction.

The tau MT-binding ability is strongly dependent on tau phosphorylation status (Ballatore et al., 2007). Several tau kinases have been identified (Hanger et al., 2009) including 5′AMP-activated protein kinase (AMPK) (Thornton et al., 2011), a heterotrimeric Ser/Thr protein kinase complex composed of three highly conserved subunits: the catalytic α subunit (α1 or α2) and two regulatory subunits (one each of β1 or β2 and γ1-3) (Carling, 2004).

First identified as a sensor of energy status able to maintain cellular energy homeostasis (Hardie, 2011a; Poels et al., 2009), AMPK is essential for cellular survival in most mammalian cells including neurons (Metcalf et al., 2012). In the brain, AMPK activity is increased during energy stress (Berger et al., 2006; Gadalla et al., 2004). However, activated AMPK (p-AMPK) is abnormally accumulated in tauopathies: neurodegenerative diseases associated with the pathological aggregation of tau (Vingtdeux et al., 2011). Indeed, previously instances of AMPK activation have been reported to be detrimental to neuronal survival, suggesting that inhibition, rather than activation, of AMPK might be neuroprotective (McCullough et al., 2005). For example, it has been shown that downregulation of AMPK activity is beneficial in both *in vitro* and *in vivo* models of familial amyotrophic lateral sclerosis, as well as in an *in vivo* mutant TDP-43 model of motor neuron disease (Lim et al., 2012). In addition, recent studies implicate AMPK activity in the pathogenesis of Alzheimer's disease (AD) as a regulator of both tau phosphorylation and amyloidogenesis (Thornton et al., 2011; Vingtdeux et al., 2011). More recently, Domise et al. (2016) assessed the contribution of AMPKα2 activation in an *in vivo* model (mouse primary neurons) where they reported an increase of tau phosphorylation at multiple sites, whereas AMPK inhibition led to a rapid decrease of tau phosphorylation. Furthermore, in a mouse model of AD it has been demonstrated that AMPK mediates the synaptotoxic effects of Aβ42 oligomers via phosphorylating tau at Ser-262 (Guthrie et al., 2011), leading to spine loss in both hippocampal and cortical neurons. Interestingly, Ser-262 is a key residue required for tau-MT binding (Tseng et al., 2008) and, when phosphorylated, a mediator of tau toxicity in a transgenic *Drosophila* model of AD (Iijima et al., 2010). Several studies have suggested that AMPK is also required for macroautophagy (hereafter referred to as autophagy). Autophagy is a process in eukaryotic cells in which regions of cytoplasm, insoluble protein aggregates, damaged mitochondria, or invading pathogens are engulfed by a double membrane to form vesicles, fusing with lysosomes to degrade the contents (Hardie, 2011b).

AMPK mediates autophagy activation directly by phosphorylation of the protein kinase that initiates autophagy, ULK1 (Egan et al., 2011; Kim et al., 2011) and indirectly, through inactivation of the mTOR complex-1 by phosphorylating both TSC2 (Inoki et al., 2003) and Raptor (Hardie, 2008).

A decrease in autophagic activity has been linked to several neurodegenerative disorders associated with the accumulation of misfolded protein aggregates, while increased autophagy has been shown to facilitate the clearance of aggregation-prone proteins and promote neuronal survival in mouse and fly models of tauopathies (Berger et al., 2006; Caccamo et al., 2010; Majumder et al., 2011; Rodríguez-Navarro et al., 2010).

The apparent separate roles of AMPK in both promoting tau phosphorylation and regulating autophagy are therefore important to analyse. They will inform how any future potential AMPK-directed pharmacological interventions might succeed.

In the present study, we have attempted to identify the cascade of events mediated by AMPK that affect tau-induced toxicity using both a mammalian cell line and an *in vivo Drosophila* model of tauopathy. *Drosophila* has been widely used as an *in vivo* model for tauopathies, reiterating many features of the mechanisms underlying human tau-induced neurodegenerative disease, in particular the roles of tau solubility and phosphorylation (Gistelinck et al., 2012). The *Drosophila* compound eye, allowing easy access, represents the most commonly used read-out to evaluate toxicity of neurodegenerative proteins, including tau. Indeed, targeted expression of either wild-type or mutant tau in the retina caused alterations in external eye structures, characterised by size reduction and rough appearance (Prüßing et al., 2013). The *Drosophila* eye tauopathy model thus constitutes a genetically sensitised system that allows the identification of modifier mechanisms by assessing its roughening as a quantitative readout of neurotoxicity (Caudron et al., 2014). Additionally, *Drosophila* has proven to also be a powerful multicellular model for genetic and pharmacological manipulation of autophagy (Nezis, 2012).

Here we report an *in vitro* physical interaction of tau with AMPK and with active p-AMPK, and we demonstrate how AMPK modulation affects the phospho-status and stability of tau. Furthermore, *in vivo*, by overexpressing an AMPK constitutively active (AMPKα1-CA) mutation, we exacerbate tau toxicity by increasing tau phosphorylation without corresponding autophagy activation. Interestingly, treatment with the autophagy activator rapamycin was able to rescue the additional toxicity.

Our findings support the development of new therapies targeting tau clearance via AMPK-independent autophagic induction.

## RESULTS

### Tau and AMPK physically interact *in vitro* in intact mammalian cells

**Fig. 1.**
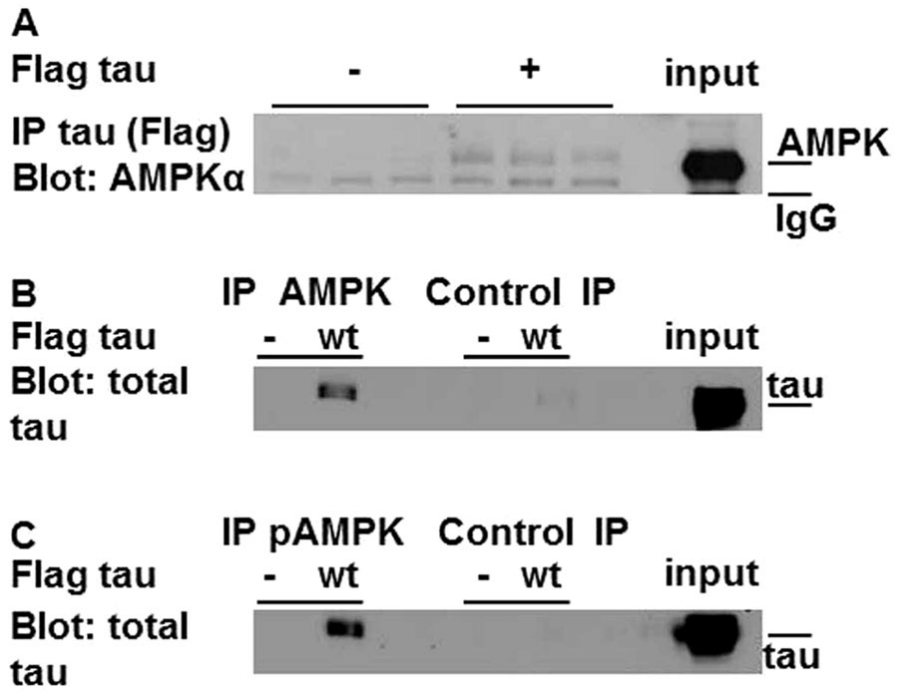
**Immunoprecipitation reveals a physical association between tau and AMPKα.** (A) Lysates from HEK293T cells transfected with a pCMV–Flag vector (negative control) and a pCMV–Flag-tau were immunoprecipitated with ANTI-FLAG M2 Affinity Gel. Immunoprecipitates were analysed by western blotting, probing against AMPKα. Uncropped blots and input controls are shown in Fig. S1A,B. (B) Identical lysates were immunoprecipitated with anti-AMPKα antibody and then incubated with protein A-Sepharose. Immunoprecipitates were analysed by western blotting, probing against total tau. Control blots are shown in Fig. S1C. (C) Interaction of tau with active AMPK (pAMPKα). Identical lysates were immunoprecipitated with anti-phospho-AMPKα (Thr172) antibody and then incubated with protein A-Sepharose. Immunoprecipitates were analysed by western blotting, probing against total tau. Control blots are shown in Fig. S1D.

It has been demonstrated that activated AMPK phosphorylated at Thr-172 (p-AMPK) is abnormally accumulated in all the major tauopathies, including AD (Vingtdeux et al., 2011). To determine if AMPK and tau are able to physically interact, we chose a simple mammalian cell-line model (HEK293T cells). This cell line does not express tau (Guthrie et al., 2011) and has been previously used to study AMPK-regulated events, including autophagy (Kim et al., 2011). Additionally, it has recently been proposed as a non-neuronal cell model where tau pathology can be studied in a stable, viable and easily transfected human cell line (Houck et al., 2016). N-terminal FLAG-tagged tau was transfected into HEK293T cells and immunoprecipitation of tau was performed with anti-FLAG antibody. Detection of co-immunoprecipitating species was performed by western blotting with an anti-AMPKα antibody. Tau was found to co-immunoprecipitate with endogenous AMPKα (Fig. 1A). Further validation for AMPK/tau interaction was provided by immunoprecipitating endogenous AMPK with an anti-AMPKα antibody, whilst detecting the co-immunoprecipitating species with an anti-tau antibody (Fig. 1B). Similarly, by immunoprecipitating with an anti-phospho antibody against AMPKα (p-AMPKα) we found that tau also physically interacts with active AMPKα (Fig. 1C).

### AMPKα1 affects exogenous tau stability *in vitro*

It has previously been demonstrated that AMPK can phosphorylate tau at Ser 262 in a cell-free *in vitro* assay (Vingtdeux et al., 2011), as well as by reintroducing the AMPK upstream activator LKB1 in to HeLa cells (Guthrie et al., 2011). We now wished to determine the consequence of AMPK activity on tau phospho-status in our HEK293T intact cell model. To evaluate the effect of AMPK overactivation on tau, we used a constitutively active form of AMPKα1 (myc-tagged AMPKα1-CA). Alternatively, to evaluate the effect of reduced AMPK activity on tau, we used a kinase-dead (K45R) variant of the AMPKα1 subunit (AMPKα1-KD), previously demonstrated to act as a dominant-negative inhibitor of endogenous AMPK (Crute et al., 1998). As reported in COS7 cell lines (Crute et al., 1998), transfection of AMPKα1-KD in to HEK293T cells led to a significant degradation of endogenous AMPK protein (Fig. S2A, and quantification Fig. S2B).

**Fig. 2.**
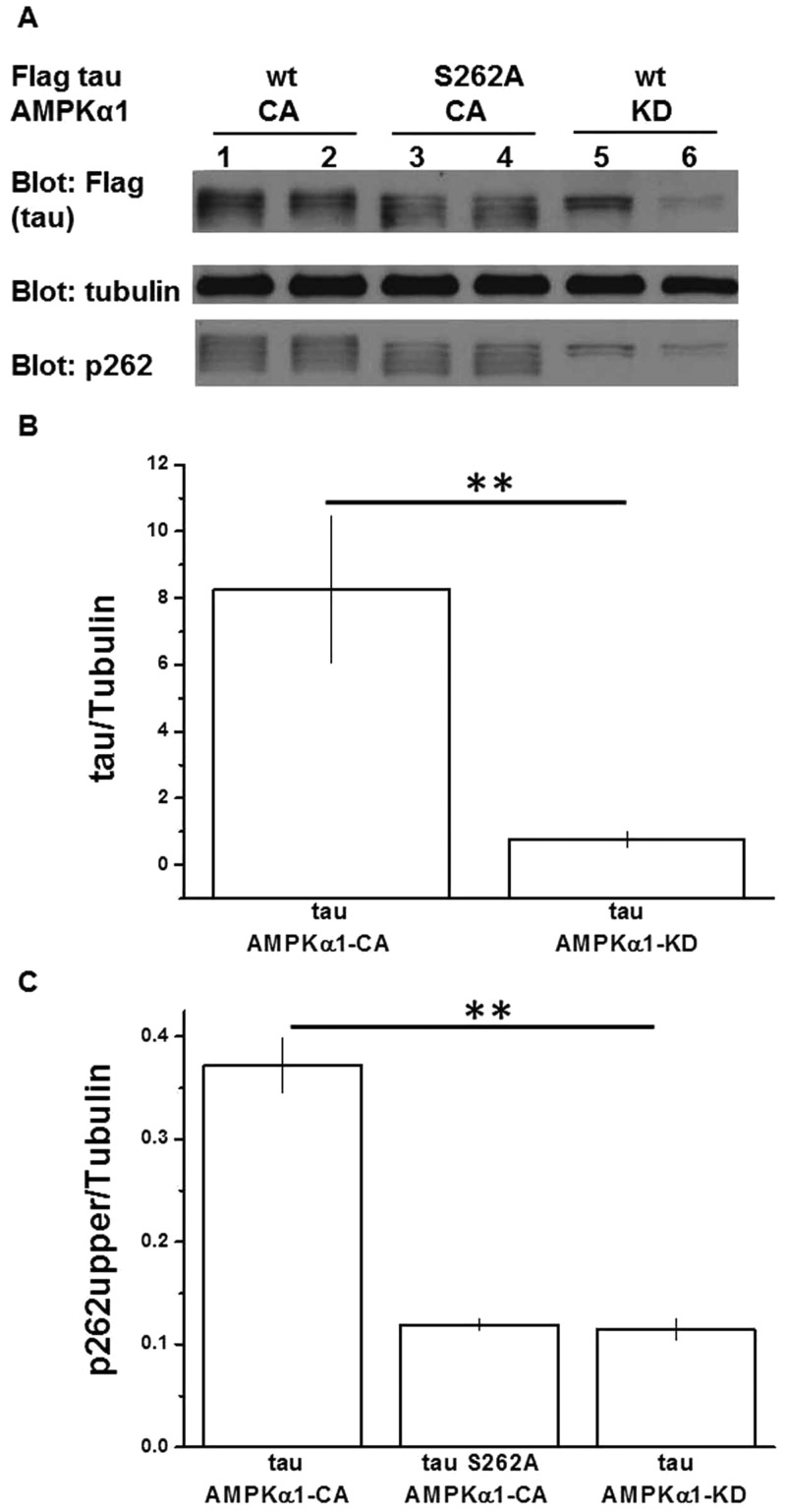
**AMPKα1 affects exogenous tau stability.** (A) Extracts from HEK293T transfected as indicated were used for detection of flagged-tau and phospho-tau, probing with anti-Flag and anti-p262-tau antibodies respectively, and then normalised with respect to tubulin. (B) Tau stability quantification expressed as a ratio between tau/tubulin is shown in the graph. Data are shown as mean±s.d.; 8.25±2.2 for tau wt+AMPKα1-CA, 0.77±0.24 for tau wt+AMPKα1-KD. *P* values were calculated by one-way ANOVA. Asterisks indicate significant differences (*P*<0.05). **P<0.01, *n*≥3 independent HEK293T transfections. (C) Quantification of phospho-tau species as a ratio between p262 tau/tubulin is shown in the graph. Data are shown as mean±s.d.; 0.37±0.03 for tau wt+AMPKα1-CA, 0.12±0.005 for tau S262A+AMPKα1-CA, 0.11±0.01 for tau wt+AMPKα1-KD. *P* values were calculated by one-way ANOVA. Asterisks indicate significant differences (*P*<0.05). **P<0.01, *n*≥3 independent HEK293T transfections.

Knockdown of endogenous AMPK via AMPKα1-KD overexpression caused dramatic reductions in total tau soluble fraction levels (an 80% reduction, Fig. 2A lanes 5-6, and quantification Fig. 2B) compared to AMPKα1-CA-transfected cells (Fig. 2A lanes 1-2), with a concomitant loss of some tau species.

In an attempt to identify the phosphorylation status of the different tau species detected, we co-transfected AMPKα1-CA and FLAG-tagged tau S262A. Western blots of cells transfected with this mutant tau (Fig. 2A lanes 3-4) revealed that it migrated differently to the wild-type tau. Tau S262A protein displayed, as expected for a non-phosphorylatable mutant, a distinct migration pattern characterised by a faster mobility compared with the wild-type tau (Fig. 2A lanes 1-2). In particular, the tau S262A migration pattern allowed us, by comparison with the wild-type tau migration pattern, to identify and quantify (Fig. 2C) the tau species phosphorylated at Ser-262. Interestingly, the lost species in AMPKα1-KD-transfected cells correspond to the Ser-262 phosphorylated species (Fig. 2A lanes 5-6). Thus, the genetic inactivation of endogenous AMPK, via AMPKα1-KD, results in a lack of Ser-262 phosphorylated tau, as well as the loss of other species yet unidentified. This suggests that under normal conditions, the phosphorylation of tau by AMPK is an important determinant of tau stability and that Ser-262 is one of the sites involved in this process.

### AMPK interacts with tau *in vivo*

#### *In vivo* interactions between human tau and *Drosophila* AMPK

**Fig. 3.**
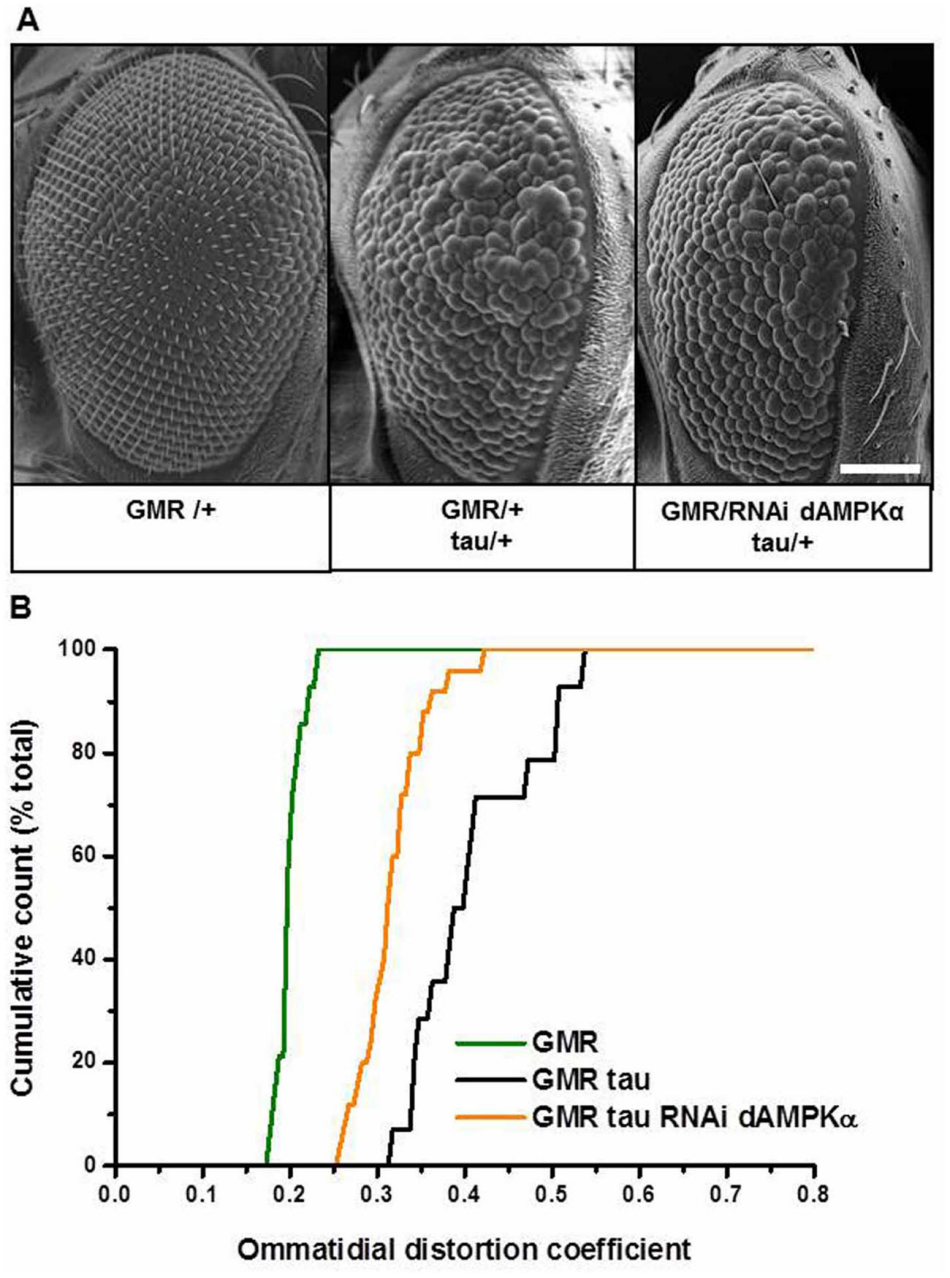
**Downregulation of *Drosophila* AMPKα (dAMPKα) partially rescued the tau-induced degenerative eye phenotype.** (A) Scanning electron microscope (SEM) images of the external eye of control flies (GMR/+), flies expressing tau (GMR/+; tau/+) and flies co-expressing tau and RNAi dAMPKα (GMR/+; tau/ RNAi dAMPKα) under the control of the GMR-GAL4 driver. Scale bar: 100 µm. (B) Quantitative analysis of tau-induced toxicity displayed as the cumulative distribution of ommatidial distortion coefficients (DC) obtained via QED analysis for control flies (GMR, green line), flies expressing tau (GMR tau, black line) and flies co-expressing tau and RNAi dAMPKα (GMR tau RNAi dAMPKα, orange line) under the control of the GMR-GAL4 driver. Co-expression of RNAi dAMPKα with tau reduced tau-induced ommatidial distortion, as indicated by the leftward shift of the DC distribution, compared to the distribution of ommatidial distortion coefficients for flies expressing tau alone. The SEM images are representative of the median DC values for each genotype. The phenotypes associated with the lower and upper DC values can be seen in Fig. S5. GMR *n*=14, GMR tau *n*=14, GMR tau RNAi dAMPKα *n*=25. Levels of significance for Mann–Whitney directional tests are <0.01 for both the compared groups: GMR versus GMR tau and GMR tau versus GMR tau RNAi dAMPKα. Validation of dAMPK downregulation upon expression RNAi dAMPKα under the control of the GMR-GAL4 driver can be seen in Fig. S3B.

While HEK293T cells have been useful to determine the biochemical interactions of AMPK and tau within an intact cellular system, they are not representative of either neurons or an *in vivo* situation. *Drosophila melanogaster* models of tau toxicity have been widely used to investigate interactions. Using the binary system UAS-GAL4 (Brand and Perrimon, 1993), it has been shown that misexpression of wild-type human tau in the fly, under the control of the retina-specific glass multiple repeat enhancer (GMR) (Freeman, 1996), caused a moderate rough eye phenotype (Wittmann et al., 2001). The tau-induced phenotype thus provides an excellent readout with which to assay tau toxicity (Jackson et al., 2002). In order to investigate whether AMPKα is a mediator of tau-dependent toxicity, we genetically downregulated endogenous *Drosophila* AMPKα using RNA interference (RNAi dAMPKα) under the control of the retina-specific GMR driver. The presence of the hemizygous GMR driver (Fig. 3A) or the expression of RNAi dAMPKα (data not shown) caused no appreciable visible external phenotype, indicating that expression of RNAi AMPKα is not detrimental to the *Drosophila* eye. Co-expression of tau and RNAi dAMPKα (GMR/+; tau/ RNAi dAMPKα) partially rescued the tau-induced eye phenotype as determined by scanning electron microscopy (Fig. 3A).

To provide a quantitative evaluation of the eye phenotype, we used a statistical procedure, Quantitative Edge Detection (QED), for the assessment of ommatidial distortion in terms of deviation from ommatidial roundness caused by transgenic manipulations of the eye (Caudron et al., 2014). The distortion coefficient (DC) ranges from 0 for no distortion to 1 for maximal distortion. Comparison of scanning electron microscope (SEM) images from GMR/+ and GMR/tau-expressing eyes showed that the distribution of ommatidial distortion was significantly different between the two, as quantified by the mean DC values (±s.d.; 0.201±0.015, *n*=15 and 0.409±0.070, *n*=15, respectively; Fig. 3B green and black line, respectively). QED analysis confirmed the reduced severity of the GMR/tau/RNAi dAMPK genotype compared with the GMR/tau alone, seen upon visual examination of the SEMs, via a leftward shift in the cumulative distribution of DC (mean DC=0.317±0.037, *n*=25; respectively, Fig. 3B orange line).

#### AMPK enhances tau neurotoxicity in a *Drosophila* model

In order to recapitulate the human AMPK/tau pathway in our *in vivo Drosophila* model, we co-expressed human tau with the relevant human AMPKα1 mutants previously characterised *in vitro* and assayed the resultant toxicity. Several insertion lines for both transgenes were used for the initial experiments; the results for two lines of the human AMPKα1-CA transgene (hereafter AMPKα1-CA) and three lines of the human AMPKα1-KD transgene (hereafter AMPKα1-KD) were used for initial SEM analyses and similar results were obtained for each transgene (data not shown). The overexpression of the AMPKα1 transgenes under the control of the GMR-GAL4 driver in the resulting AMPKα1-CA and AMPKα1-KD lines were validated by western blot (Fig. S3A,B). The overexpression of those transgenes caused no appreciable external eye phenotype (Fig. S4A,B).

**Fig. 4.**
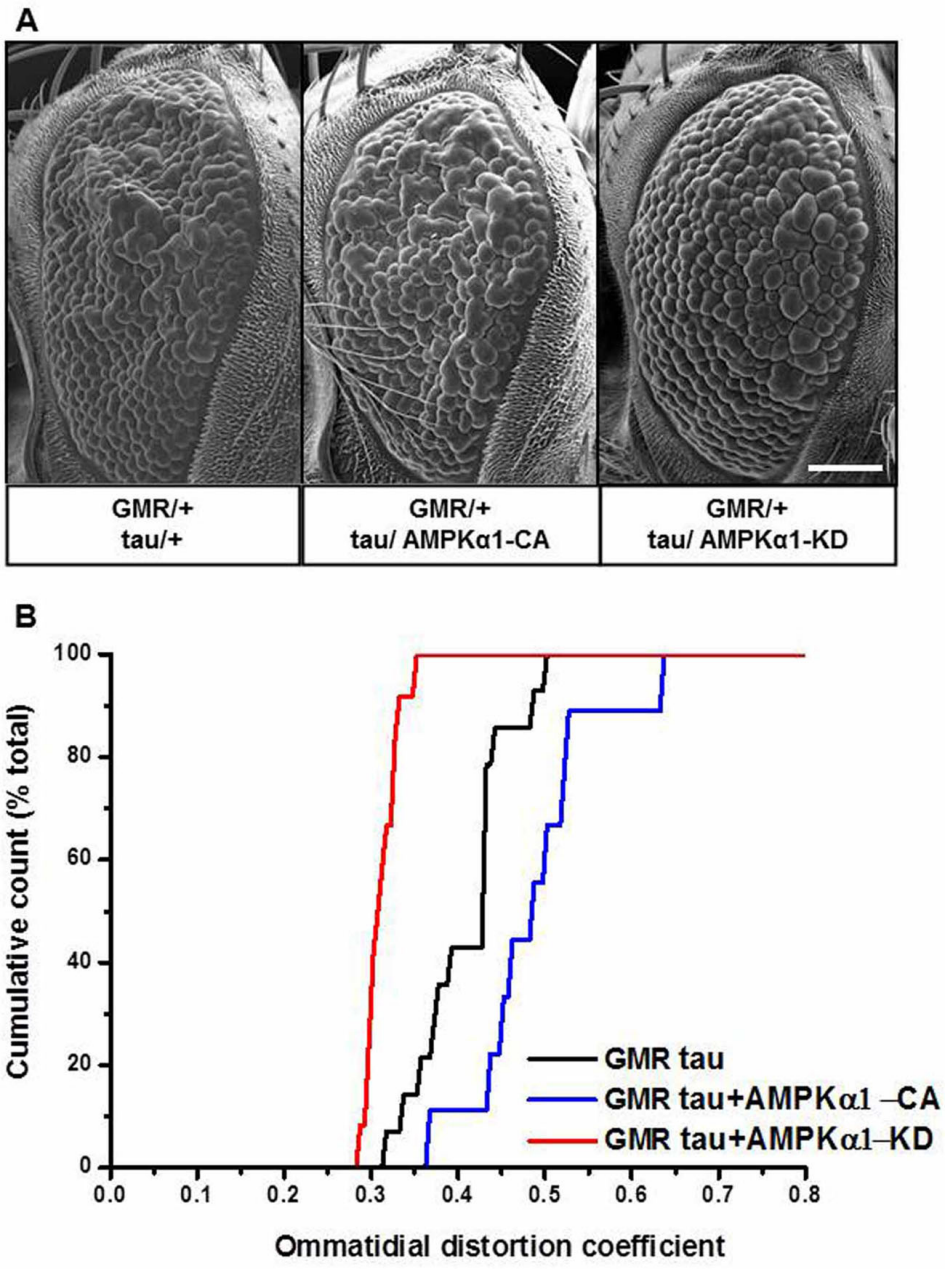
**Recapitulation of the human AMPK/tau pathway in the *Drosophila* eye.** (A) Scanning electron microscope (SEM) images of the external eye of flies expressing tau (GMR/+; tau/+) and flies co-expressing tau and human AMPKα1-CA (GMR/+; tau/ AMPKα1-CA) or tau and human AMPKα1-KD (GMR/+; tau/ AMPKα1-KD) under the control of the GMR-GAL4 driver. Scale bar: 100 µm. (B) Quantitative analysis of tau-induced toxicity as distribution of ommatidial distortion coefficients (DC) for flies expressing tau (GMR tau, black line), flies co-expressing tau and AMPKα1-CA (GMR tau+ AMPKα1-CA, blue line), tau and AMPKα1-KD (GMR tau+ AMPKα1-KD, red line) under the control of the GMR-GAL4 driver. The SEM images are representative of the median DC values for each genotype. The phenotypes associated with the lower and upper DC values, can be seen in Fig. S6. GMR tau *n*=14, GMR tau+ AMPKα1-CA *n*=9, GMR tau+ AMPKα1-KD *n*=12. Levels of significance for Mann–Whitney directional tests are <0.01 for both the compared groups: GMR tau versus GMR tau+ AMPKα1-CA and GMR tau versus GMR tau+ AMPKα1-KD. Validation of AMPKα1 overexpression in transgenic lines AMPKα1-CA and AMPKα1-KD under the control of the GMR-GAL4 driver can be seen in Fig. S3A,B.

Co-expression of tau and AMPKα1-CA (GMR/+; tau/AMPKα1-CA) caused an exacerbation of the toxic tau eye phenotype (Fig. 4A). By using QED, we found a significant shift to the right of the DC distribution and an increase in the mean DC when compared to both control and tau alone (0.488±0.073, *n*=10; Fig. 4B, blue line).

In contrast, co-expression of tau and AMPKα1-KD (GMR/+; tau/AMPKα1-KD) ameliorated the basal tau phenotype. Quantitative analysis using QED validated the SEM analyses (mean DC=0.314±0.018, *n*=12) and the rescue resulted in a shift to the left of the DC distribution compared with tau alone (Fig. 4B red line and black line, respectively). These data are consistent with the hypothesis that AMPK activation can promote tau toxicity in this model.

#### AMPKα1 overactivation promotes exogenous tau phosphorylation *in vivo*

**Fig. 5.**
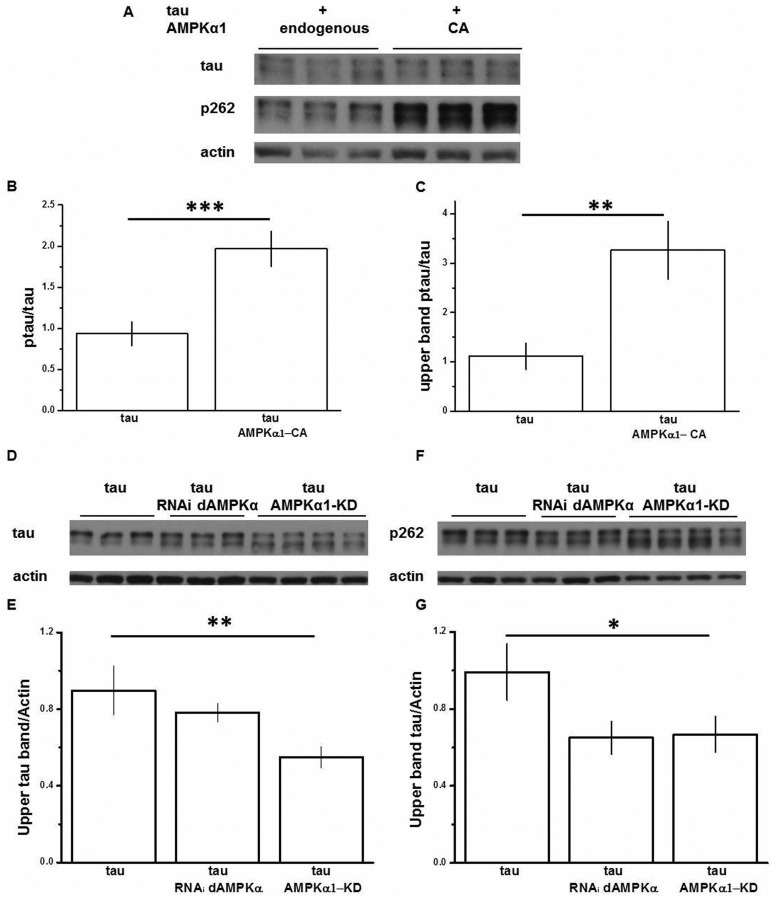
**AMPKα1 modulation affects tau phosphorylation and stability.** AMPKα1 overactivation promotes tau phosphorylation. (A) Extracts of fly heads from flies expressing tau alone and in combination with AMPKα1-CA probed with total tau or pS262 antibodies and normalised against actin. (B) Quantification of tau phosphorylation as a ratio between ptau/total tau is shown in the graph. Data are shown as mean±s.d.; 0.94±0.14 for tau wt and 1.97±0.21 for tau+ AMPKα1-CA. *P* values were calculated by one-way ANOVA. Asterisks indicate significant differences (*P*<0.05). ***P<0.001, *n*≥3 independent preparations. (C) Quantification of phospho-tau species as a ratio between the ptau slower mobility upper band (upper band ptau)/total tau is shown in the graph. Data are shown as mean±s.d.; 1.11±0.26 for tau and 3.26±0.59 for tau+AMPKα1-CA. *P* values were calculated by one-way ANOVA. Asterisks indicate significant differences (*P*<0.05). **P<0.01, *n*≥3 independent preparations. AMPKα1 downregulation destabilises pathological tau species and changes its phosphorylation pattern. (D) Extracts of fly heads from flies expressing tau alone and in combination with RNAi dAMPKα or AMPKα1-KD, probed with total tau antibody and normalised against actin. (E) Quantification of pathological tau species as a ratio between the slower mobility upper band/actin is shown in the graph. Data are shown as mean±s.d.; 0.90±0.13 for tau, 0.78±0.05 for tau+RNAi dAMPKα, 0.55±0.06 for tau+AMPKα1-KD. *P* values were calculated by one-way ANOVA. Asterisks indicate significant differences. **P<0.01, *n*≥3 independent preparations. (F) Extracts of fly heads from flies expressing tau alone and in combination with RNAi dAMPKα or AMPKα1-KD, probed with total phospho-tau (p262) antibody and normalised against actin. (G) Quantification of pathological phospho tau species as a ratio between the slower mobility upper band/actin is shown in the graph. Data are shown as mean±s.d.; 0.99±0.14 for tau, 0.65±0.09 for tau+RNAi dAMPKα, 0.67±0.09 for tau+AMPKα1-KD. *P* values were calculated by one-way ANOVA. Asterisks indicate significant differences. *P<0.05, *n*≥3 independent preparations.

Phosphorylation of tau protein characterises human tauopathy disorders. To address whether AMPK overactivation-induced changes in tau toxicity coincide with changes in tau phosphorylation, we analysed tau phosphorylation in tau/AMPKα1-CA flies. Western blots analyses of fly head extracts in tau/AMPKα1-CA-expressing flies showed that AMPK plays an important role in the phosphorylation of tau: increased AMPK activity drives tau phosphorylation in the fly, as shown in Fig. 5A, where samples were probed with antibody against total tau (upper panel), against p262 (middle panel) and against actin (lower panel) and quantified (Fig. 5B). This analysis revealed that tau in tau/AMPKα1-CA flies is strongly phosphorylated in comparison to the flies expressing tau alone (Fig. 5B).

Probing our samples with antibodies against total or phospho tau, we observed that tau protein runs with a specific electrophoretic profile, characterised by a strong ∼60 kDa band and an additional lower band (∼55 kDa). Previous work has shown that pathologically hyperphosphorylated (including pSer-262) human tau band runs at ∼60 kDa, whereas the normally phosphorylated tau runs in the range of 55–60 kDa (Ali et al., 2012; Le Corre et al., 2006). Consistent with this observation, AMPK overactivation caused an increase in the immunoreactivity for the pathologically hyperphosphorylated species, as showed in Fig. 5C where we quantified the immunoreactivity for p262 of the upper mobility band.

#### AMPKα downregulation destabilises pathological tau species and changes tau phosphorylation pattern

To address whether the reduced tau toxicity associated with AMPK downregulation (Fig. 4B) coincides with changes in tau protein phosphorylation, we analysed tau protein in both tau/dAMPK RNAi and tau/AMPKα1-KD flies. Analysis of western blots showed that as in HEK293T, AMPK plays an important role in the stability of tau as downregulation of AMPK activity, both via RNAi and AMPKα1-KD, and drives tau protein destabilisation (Fig. 5D). Interestingly, AMPK downregulation caused a shift to the higher mobility band and a reduction in the immunoreactivity for the pathologically hyperphosphorylated species, as showed in Fig. 5E where we quantified the reduction of the slower mobility upper band.

Probing our samples with an antibody against tau phosphorylated on Ser-262 (p262), we validated that the pathologically hyperphosphorylated band consists of tau hyperphosphorylated at Ser-262 and the immunoreactivity for that band is reduced by AMPK downregulation (Fig. 5F,G). This is again supportive of a link between AMPK downregulation and neuroprotection.

#### The catalytic subunit of AMPK influences autophagy in the *Drosophila* eye.

It has been demonstrated that Ref(2)P, the homologue of mammalian p62, is a major component of protein aggregates formed during normal aging in the adult fly brain. It also accumulates in protein aggregates in flies that are defective in autophagy and in models of human neurodegenerative diseases (Nezis et al., 2008). Accordingly increases in Ref(2)P are interpreted as reflecting dysfunctional autophagy.

Given AMPK's known roles in autophagy activation, we sought to test the autophagy status in our transgenic fly models. We therefore assayed Ref(2)P accumulation in tau, tau/AMPKα1-CA and tau/AMPKα1-KD flies head extracts.

**Fig. 6.**
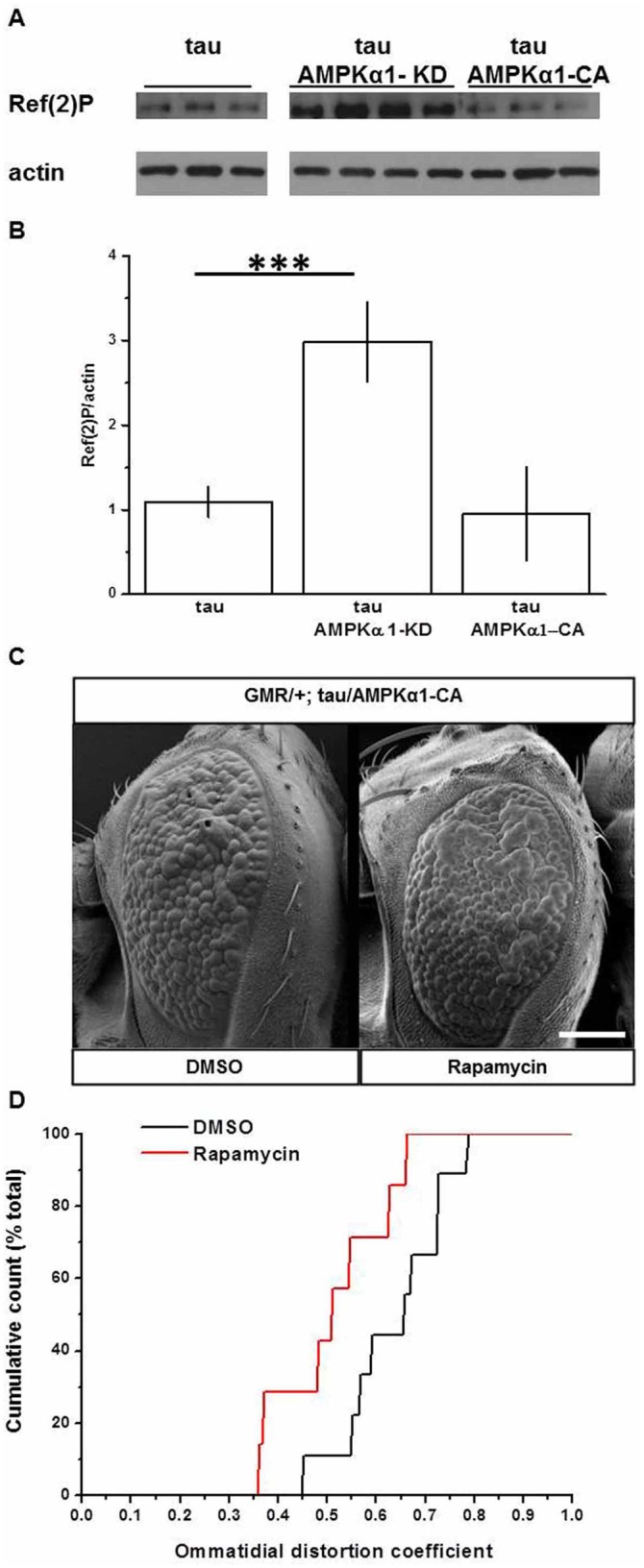
**Modulation of AMPK, autophagy and AMPKα1-CA tau-mediated toxicity.** Modulation of AMPK and Ref(2)P accumulation in tau flies. (A) Western blot analysis of fly head lysates of tau, tau/AMPKα1-KD and tau/AMPKα1-CA flies probed with anti-Ref(2)P antibody and normalised for actin. Lysates from tau (control), tau/AMPKα1-KD and tau/AMPKα1-CA (experiments) were loaded on the same gel and probed on the same membrane. (B) Quantification of Ref(2)P accumulation as a ratio between Ref(2)P/actin is shown in the graph. Data are shown as mean±s.d.; 1.09±0.18 for tau, 2.98±0.48 for tau/AMPKα1KD and 0.95±0.57 for tau/AMPKα1-CA. *P* values were calculated by one-way ANOVA. Asterisks indicate significant differences. ***P<0.001, *n*≥3 independent preparations. Rapamycin treatment ameliorates AMPKα1-CA mediated tau phenotype. (C) SEM images of the external eye of control (DMSO) and rapamycin-treated (Rapamycin) flies co-expressing tau and AMPKα1-CA (GMR/+; tau/AMPK CA) under the control of the GMR-GAL4 driver. Scale bar: 100 µm. (D) Quantitative analysis of tau-induced toxicity as a distribution of ommatidial distortion coefficients (DC) for flies co-expressing tau and AMPKα1-CA (GMR/+; tau/AMPK CA) fed with DMSO (black line) and rapamycin (red line). The SEM images are representative of the median DC values for each genotype. The phenotypes associated with the lower and upper DC values can be seen in Fig. S7. DMSO *n*=9, Rapamycin *n*=7. Level of significance for a Mann–Whitney directional test is <0.01.

In our model, we detected no changes in Ref(2)P accumulation in tau/AMPKα1-CA compared to tau alone (Fig. 6A, quantification Fig. 6B), demonstrating that overexpression of AMPKα1-CA, which caused an exacerbation of tau-dependent toxicity, did not affect autophagy activation. In contrast, Ref(2)P accumulated in tau/AMPKα1-KD flies (Fig. 6A,B) compared to tau alone. This suggests that the downregulation of endogenous AMPK impairs autophagy.

#### Pharmacological activation of autophagy by rapamycin decreases AMPKα1-CA tau-mediated toxicity

In order to test whether induction of autophagy downstream of AMPKα1-CA was beneficial in AMPKα1-CA-mediated tau-dependent toxicity, we fed rapamycin to flies co-expressing tau and human AMPKα1-CA (tau/AMPKα1-CA) and analysed the eye phenotypes. Whereas 0.1 µM rapamycin had no effect (data not shown), 1 µM treatment ameliorated the tau AMPKα1-CA mediated exacerbation of the toxic tau eye phenotype, compared with the same genotype fed with DMSO (Fig. 6C). QED quantitative analysis validated the SEM analyses (DC=0.64±0.10, *n*=9 for DMSO control flies versus 0.51±0.11, *n*=7 for rapamycin-treated flies) and the rescue resulted in a clear shift to the left of the DC distribution compared with DMSO-treated flies (Fig. 6D black line and red line, respectively). Collectively, these data are consistent with the hypothesis that induction of autophagy downstream of AMPK-mediated tau phosphorylation is beneficial in reducing tau-dependent neurodegeneration.

## DISCUSSION

The exact role of AMPK in neurodegeneration is still highly controversial. It has been reported that a beneficial outcome of AMPK activation is to promote neuronal maintenance under normal conditions and upon metabolic stress. Studies in *Drosophila* have strengthened this notion demonstrating that AMPK deficiency causes neurodegeneration (Poels et al., 2009; Ronnett et al., 2009).

However, AMPK activation has also been found to be detrimental to neuronal survival (Li et al., 2006; McCullough et al., 2005). Downregulation of AMPK has been reported to be beneficial in models of familial amyotrophic lateral sclerosis, as well as in an *in vivo* model of motor neuron disease (Lim et al., 2012). In addition, recent studies implicate AMPK activity in the pathogenesis of AD as a regulator of both tau phosphorylation and amyloidogenesis (Thornton et al., 2011; Vingtdeux et al., 2011), and the contribution of AMPKα activation has been assessed in mouse primary neurons where an increase of tau phosphorylation at multiple sites upon endogenous AMPK activation has been reported, whereas AMPK inhibition led to a rapid decrease of tau phosphorylation (Domise et al., 2016).

Among its different roles, AMPK activates autophagy (Egan et al., 2011; Hardie, 2008; Inoki et al., 2003; Kim et al., 2011), the main pathway for eukaryotic cells to degrade organelles and long-lived proteins (Jaeger and Wyss-Coray, 2009). Interestingly, defective autophagy has also been implicated in the pathogenesis of several major neurodegenerative diseases, particularly AD (Nixon and Yang, 2011). Additionally, defects in autophagy lead to an increased accumulation of tau phosphorylated at the KXGS motifs (Wang et al., 2009) such as Ser-262, a species also reported to be less susceptible to proteasomal degradation mediated by Hsp90 (Dickey et al., 2006, 2007).

In the present study, we assessed in a *Drosophila* *in vivo* model the contribution of AMPK modulation to tau-mediated toxicity.

We initially established whether AMPK physically interacts with tau in HEK293T cells. Using immunoprecipitation, we found that AMPK can interact with tau (Fig. 1A,B) as can the active form of AMPK (Fig. 1C). Attempting to define the simplest and most effective way to increase AMPK activity, we took advantage of the AMPKα1-CA mutation. This mutation frees the catalytic α-subunit from its regulatory β and γ subunits (Woods et al., 2000) rendering it constitutively active. Using a *Drosophila* model expressing human tau in the developing fly eye we examined *in vivo* the effect of AMPKα1-CA on tau toxicity. In these flies we exacerbated tau toxicity as determined by analysis of ommatidial distortion (Fig. 4). Comparative analysis of protein lysates from flies overexpressing tau±AMPKα1-CA showed that co-expression of AMPKα1-CA significantly elevated the immunoreactivity of pSer-262 (Fig. 5A).

Previously, in a cell-free *in vitro* assay, AMPK has been demonstrated to phosphorylate tau at Ser-262 (Vingtdeux et al., 2011). Phosphorylation at Ser-262 is significantly elevated in human AD pre- and intra-neuronal tangles (Augustinack et al., 2002). Consequently, phosphorylation of tau at Ser-262 by AMPK may represent an important event in the early stages of AD pathogenesis.

It has been proposed that tau phosphorylated at such KXGS motifs is likely to be resistant to proteasomal degradation (Dickey et al., 2006, 2007) and to accumulate when autophagy is defective (Wang et al., 2009). Indeed, in some of our lines, we found that AMPKα1-CA overexpression resulted in increased exogenous tau stability (data not shown).

We have also demonstrated that downregulating AMPK activity affects tau in both *in vitro* and *in vivo* models. In fact, we established that when endogenous AMPK activity is decreased using dominant-negative AMPKα1-KD in HEK293T cells, soluble tau levels also decrease. This relationship was particularly evident in pathologically phosphorylated species (Fig. 2A lane 5-6, Fig. 2B). In the fly, we downregulated dAMPK by using two independent means: dAMPK-RNAi-mediated silencing, and by overexpressing the human AMPKα1-KD. In both cases, by reducing endogenous dAMPK, we reduced the tau-induced toxicity (Figs 3 and 4).

Particularly, the diminished tau toxicity is likely due to a reduction in both tau phosphorylation and stability: lowering AMPK *in vitro* via expression of AMPKα1-KD altered the phosphorylation profile of tau and led to compromised soluble tau stability in cells (Fig. 2). *In vivo*, analysis of protein lysates from flies expressing tau (Fig. 5) revealed the presence of a distinct doublet, with one band having a higher electrophoretic mobility. This indicates the presence of a population of tau species at a different phosphorylation status, which results in different electrophoretic migration.

It has been proposed (Ali et al., 2012; Le Corre et al., 2006) that pathologically hyperphosphorylated (including pSer-262) human tau bands run at 64 kDa, whereas the normally phosphorylated tau runs in the range of 55–60 kDa. Interestingly, in tau±RNAi dAMPK or AMPKα1-KD flies we observed how the reduction of AMPK activity affects the levels and distribution of tau proteins (Fig. 5D,F). Specifically, reduced AMPK levels decreased the overall levels of pathological tau high molecular species, resulting in a band shift in the migration of total (Fig. 5D) and pSer-262 (Fig. 5F) tau species. In addition, the marginal but still detectable immunoreactivity of pathological tau high molecular weight species phosphorylated at Ser-262 is a clear indication that Ser-262 is not the only site phosphorylated in the pathological tau and the remaining immunoreactivity could be responsible for the residual toxicity in tau±RNAi dAMPK or AMPKα1-KD fly.

Our data are consistent with a detrimental effect of AMPK overactivation on tau-mediated toxicity. In our AMPK overactivation model we detected more toxic hyperphosphorylated tau. On the other hand, AMPK downregulation, preventing tau phosphorylation, destabilised the pathological tau species and rescued the tau toxic eye phenotype in the fly.

How might this be explained with the observations of the direct (Egan et al., 2011; Kim et al., 2011) and indirect (Hardie, 2008, 2011b) activation of autophagy by AMPK and the attenuated tauopathy reported in different models after autophagy induction (Congdon et al., 2012; Schaeffer et al., 2012)?

**Fig. 7.**
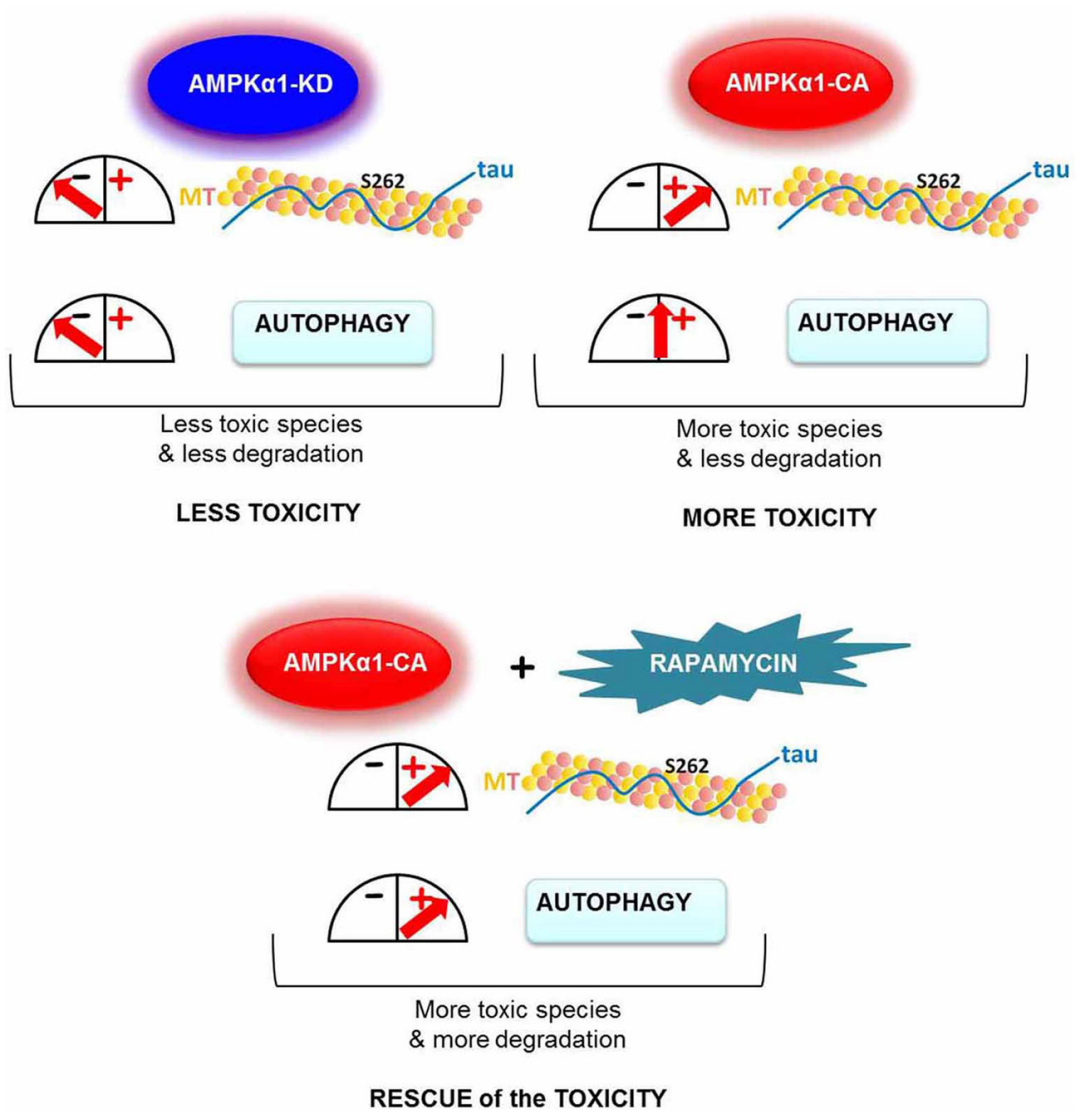
**AMPK-mediated tau phosphorylation and autophagy activation: model of action.** Downregulation of AMPK via AMPKα1-KD, although causing a reduction in the activation of autophagy, resulted in less tau toxicity, likely due to the reduction of tau phosphorylation and hence less toxic tau species. In contrast, overexpression of AMPKα1-CA results in increased tau toxicity due to AMPKα1-CA's ability to phosphorylate tau, thereby promoting the appearance of toxic tau species, without affecting autophagy, which would otherwise degrade such toxic species. Finally, overexpression of AMPKα1-CA concomitant with pharmacological induction of autophagy, via rapamycin, resulted in rescued AMPKα1-CA-mediated tau toxicity.

To attempt to answer this question we measured the levels of accumulation of Ref(2)P, the *Drosophila* orthologue of p62, a component of protein aggregates in flies defective in autophagy and models of human neurodegenerative diseases, in which it strongly accumulates (Nezis et al., 2008). This approach allowed us to assess in our different experimental conditions the level of AMPK-mediated autophagy activation and to correlate that with the tau toxicity. As schematised in Fig. 7, our data demonstrates that downregulation of AMPK activity via AMPKα1-KD showed an accumulation of Ref(2)P (Fig. 6). This is indicative of a reduction in autophagic flux, consistent with defective autophagy, as expected from mammalian models, where the expression of a dominant-negative form of AMPK completely inhibited autophagic proteolysis in HT-29 and HeLa cells (Meley et al., 2006). From the work presented here our model demonstrates that in AMPKα1-KD-tau flies, tau does not accumulate. Such flies do not need the beneficial effect of autophagy induction since they are likely to be able to clear tau via other degradative pathways. Consequently the AMPK-mediated autophagy downregulation does not affect the tau phenotype.

Conversely, the increase in tau toxicity provoked by AMPKα1-CA is due to the increased phosphorylation and possible reduced degradation of tau, concomitant with the inability of AMPKα1-CA to effectively induce autophagy. Indeed, the lack of increased autophagic induction by AMPKα1-CA has been previously demonstrated in HT-29 and HeLa mammalian cells (Meley et al., 2006).

Eventually, the reduction in tau toxicity found in AMPKα1-CA-tau flies treated with rapamycin (Fig. 6C,D), is consistent with the beneficial outcome in autophagy induction downstream of AMPK.

The *Drosophila* model presented in this paper demonstrates an ability to affect tau toxicity via AMPK-modulation *in vivo*.

In human tauopathies, stress conditions result in AMPK overactivation and increased kinase activity, responsible for tau phosphorylation. Additionally, AD patients are known to be defective in autophagy activation (Salminen et al., 2011). Our AMPKα1-CA-tau model recapitulates this disease process by increasing tau phosphorylation, in particular at Ser-262, without corresponding autophagy activation. This site is reported to promote resistance to degradation (Dickey et al., 2006, 2007), leading to tau accumulation that requires autophagy for clearance.

While AMPK is potentially able to offer protective effects through regulation of autophagy (Egan et al., 2011; Hardie, 2008, 2011a; Kim et al., 2011), given the resulting increased phospho-tau levels as observed from the detrimental effects seen here *in vivo* for AMPK-CA, we propose that this results on balance in toxic accumulation of phospho-tau. Accordingly, our results indicate that pharmacological induction of autophagy downstream of AMPK may be beneficial in tau-dependent neurodegeneration.

Taken together our findings raise the possibility that new therapies targeted at AMPK-mediated tau phosphorylation might be effective at reducing tau toxicity. The potential for such treatments to compromise autophagy are likely to be outweighed by the benefits associated with reducing phospho-tau toxicity. This opens the possibility of combinatorial therapy with treatments targeted at AMPK-independent autophagic induction to promote tau clearance. The *Drosophila* model presented here offers an opportunity to test the development of such drugs.

## MATERIALS AND METHODS

### Cell lines and transfection

HEK293T cells were maintained in DMEM of high glucose, supplemented with 10% FBS and grown at 37°C in a humidified atmosphere with 5% CO_2_. Transfections were performed using Lipofectamine 2000 (Invitrogen) following the manufacturer's protocol with empty pCMV-FLAG, pCMV-FLAG-tau (kind gifts of Dr Calum Sutherland, University of Dundee, UK), pCMV-FLAG-S262A tau or pCMV-FLAG-T231A tau plasmids in combination with empty pcDNA3 (Invitrogen), pcDNA3 N-terminal-Myc-AMPKα1-CA (kind gift of Prof. David Carling, MRC Clinical Sciences Centre, Imperial College, London, UK). pCMV-FLAG-tau is an N-terminal Flag-tagged human microtubule-associated protein tau isoform 2 (Genbank NP_005901.2), cloned into the pCMV FLAG-vector. pCMV-Myc Tag3b AMPKα1-KD and pCMV-FLAG-S262A tau were obtained by site-directed mutagenesis respectively on pCMV-Myc Tag3b AMPKα1-wt and pCMV-FLAG-tau, via site-directed mutagenesis using a QuikChange site-directed mutagenesis kit (Stratagene, site-directed mutagenesis kit (Agilent Technologies LDA UK, Stockport, UK).

### Fly stocks

The transgenic fly line carrying the human 0N4R tau, with the four tubulin-binding domains (R) at the C-terminal region and no N-terminal insert (N), was a kind gift from Dr Mel Feany (Harvard Medical School, MA, USA) (Wittmann et al., 2001). UAS-AMPKα1-CA and UAS- AMPKα1-KD transgenic lines were obtained by directional cloning of cDNAs, into the *Drosophila* expression vector, pUAST. Transgenic flies were produced by BestGene Inc. using standard procedures. UAS-AMPK RNAi flies were obtained from the VDRC (Vienna *Drosophila* RNAi Center, Vienna, Austria; Transformant ID 106200). All other fly stocks were obtained from the Bloomington *Drosophila* Stock Center (Indiana University, IN, USA). Crosses were maintained on standard cornmeal-based *Drosophila* medium at 25°C.

### Immunoprecipitation and immunoblotting

HEK293T cells were recovered by gentle pipetting in PBS1X, centrifuged at 5000 ***g*** for 5 min in a microfuge and resuspended in 0.5 ml lysis buffer [50 mM Tris-HCl pH 7.4, 150 mM NaCl, 1 mM EDTA, 1% Triton X-100, 1 mM Sodium Orthovanadate, 50 mM Sodium Fluoride, and Complete protease inhibitor tablets (Roche)]. After sonication for 15 min and incubation on ice for 10 min, samples were centrifuged at 15700 ***g*** for 30 min. The supernatant was recovered and the protein concentration determined. Proteins were either directly analysed after electrophoresis, by western blotting (see below), or immunoprecipitated. In the second case, extracts were incubated under rotation for 2 h at 4°C with ANTI-FLAG M2 Affinity Gel (Sigma, A2220) according to manufacturer's instructions, or with the relevant antibody. In the latter case, Protein A-Sepharose (Generon, Slough, UK) was then added and the mixture incubated for a further 2 h at 4°C. Sepharose beads were quickly centrifuged in a microfuge (2′ at 8000 rpm) and washed three times with lysis buffer. After the final wash, the beads were resuspended in 30 µl Laemmli sample buffer, boiled 10 min at 90°C and electrophoresed through a 10% acrylamide gel. Adult flies were collected, heads dissected and homogenised in a lysis buffer containing 50 mM Tris-HCl pH 7.5, 0.1 mM EGTA, 1 mM EDTA, 1% Triton X-100, 1 mM Sodium Orthovanadate, 50 mM Sodium Fluoride, 5 mM Sodium Pyrophosphate, 0.27 M Sucrose, 0.1% β-Mercaptoethanol and complete protease inhibitor tablets (Roche). Protein content was determined using Bradford reagent (Bio-Rad). Extracts were mixed with SDS sample buffer and heated at 90°C for 10 min before being separated by 10% SDS-PAGE.

Lysates or immunocomplexes subjected to SDS-PAGE were transferred to nitrocellulose membranes (Amersham Biosciences, GE Healthcare Life Sciences, Buckinghamshire, UK). Nitrocellulose blots were incubated at room temperature for 30 min in blocking buffer [Tris-buffered saline with 0.1% Tween containing 5% bovine serum albumin (BSA) and then incubated overnight at 4°C in 5% BSA with the recommended concentrations of indicated antibodies]. After washing three times for 15 min each with Tris-buffered saline containing 0.1% Tween, the blots were incubated with horseradish peroxidase-conjugated anti-mouse or anti-rabbit IgG (Amersham Pharmacia Biotechnology, Piscataway, New Jersey, USA), followed by washing. Immunoreactive bands were visualised with enhanced chemiluminescence substrate (Pierce, Rockford, IL, USA). Developed films were scanned using a flat-bed scanner and densities of bands were measured using ImageJ software (NIH).

Membranes were immunoblotted with the following antibodies at the indicated dilutions: tau T46 (Abcam AB22261, 1:1000), tau phospho S262 (Abcam AB4856, 1:1000), tau phospho T231 (Abcam AB30665, 1:1000), AMPKα (Cell Signaling, #2532, 1:1000), Phospho-AMPKα (Thr172) (40H9) (Cell Signaling, #2535, 1:1000), anti-FLAG M2 (Sigma, F3165, 1:5000), Anti-beta Tubulin (Abcam AB6046, 1:5000), anti-actin (JLA20, Developmental Studies Hybridoma, Ames, IA, USA; 1:2000), anti-Ref(2)P (Wyers et al., 1995) (kind gifts of Dr Ioannis P. Nezis, Life Sciences, University of Warwick, UK; 1:1000).

### Scanning electron microscopy (SEM)

To analyse external eye structure using SEM, female flies were fixed overnight in 4% PFA in PBS1X at 4°C. Flies were then dehydrated through an acetone series (10%, 30%, 50%, 70%, and 90% in H_2_O), incubating on a rotator in 1 ml of solution for 15 min at room temperature (RT). Flies were transferred to 100% acetone, for at least 2 days at 4°C, and then attached to stubs using double-sided tape, sputter coated in gold for 60 s and imaged using a Zeiss Supra55 VP SEM at 150× magnification.

### Quantitative edge detection (QED)

Software for assessing the level of ommatidial disruption in adult fly eyes (QED) was developed in collaboration with the Statistics Department at Warwick University, UK (Caudron et al., 2014). For the purpose of this study, only data for the roundness measure (termed ommatidial distortion) has been used as it is a more sensitive measure, sampling hundreds of times per image as opposed to once per image for the distance and angle measures. Raw data from QED was collected and transferred to OriginLab software, version 4 (http://www.originlab.com) in order to produce cumulative plots of ommatidial distortion versus fly count (in percent of total). Significance levels were calculated with the Mann–Whitney *U*-test.

### Rapamycin treatment

Rapamycin (LC Laboratories, Woburn, MA, USA) was dissolved in DMSO (500 μM stock) and added to fly food at 0.1 μM and 1 μM. For control food DMSO alone (0.02% and 0.2%, respectively) was added.
